# Development of PCL PolyHIPE Substrates for 3D Breast Cancer Cell Culture

**DOI:** 10.3390/bioengineering10050522

**Published:** 2023-04-26

**Authors:** Caitlin E. Jackson, David H. Ramos-Rodriguez, Nicholas T. H. Farr, William R. English, Nicola H. Green, Frederik Claeyssens

**Affiliations:** 1 Materials Science and Engineering, The Kroto Research Institute, University of Sheffield, Sheffield S3 7HQ, UK; 2 Insigneo Institute for In Silico Medicine, The Pam Liversidge Building, University of Sheffield, Sheffield S1 3JD, UK; 3 Department of Orthopaedic Surgery, UC Davis Health, Sacramento, CA 95817, USA; 4 Norwich Medical School, University of East Anglia, Norwich NR3 7TJ, UK

**Keywords:** polycaprolactone, polyHIPE, tissue engineering, CAM assay, breast cancer

## Abstract

Cancer is a becoming a huge social and economic burden on society, becoming one of the most significant barriers to life expectancy in the 21st century. In particular, breast cancer is one of the leading causes of death for women. One of the most significant difficulties to finding efficient therapies for specific cancers, such as breast cancer, is the efficiency and ease of drug development and testing. Tissue-engineered (TE) *in vitro* models are rapidly developing as an alternative to animal testing for pharmaceuticals. Additionally, porosity included within these structures overcomes the diffusional mass transfer limit whilst enabling cell infiltration and integration with surrounding tissue. Within this study, we investigated the use of high-molecular-weight polycaprolactone methacrylate (PCL–M) polymerised high-internal-phase emulsions (polyHIPEs) as a scaffold to support 3D breast cancer (MDA-MB-231) cell culture. We assessed the porosity, interconnectivity, and morphology of the polyHIPEs when varying mixing speed during formation of the emulsion, successfully demonstrating the tunability of these polyHIPEs. An *ex ovo* chick chorioallantoic membrane assay identified the scaffolds as bioinert, with biocompatible properties within a vascularised tissue. Furthermore, *in vitro* assessment of cell attachment and proliferation showed promising potential for the use of PCL polyHIPEs to support cell growth. Our results demonstrate that PCL polyHIPEs are a promising material to support cancer cell growth with tuneable porosity and interconnectivity for the fabrication of perfusable 3D cancer models.

## 1. Introduction

Breast cancer is a leading cause of death for women [1], with an estimated 51,400 new diagnoses of ductal carcinoma in the US in 2022 [2]. MDA-MB-231 cell line (isolated from a patient with invasive ductal carcinoma) is a metastatic, triple-negative (ER, PR, and HER), and E-cadherin-negative breast cancer [3]. It is a cell line commonly used to model late-stage breast cancer. MDA-MB-231 cells have been shown to be invasive *in vitro* and spontaneously metastasise in *in vivo* models [4,5,6]. This is a well-defined and frequently used cell line that can be used to develop relevant 3D *in vitro* models for assessing drug efficacy, a research area that is increasingly needing further development. Currently, there is a >96% failure rate of potential drug candidates for breast cancer in clinical trials, driven by a lack of translatability of *in vitro* efficacy *in vivo* [1]. *In vitro* drug screening platforms that closely mimic *in vivo* models can reduce the use of *in vivo* animal models, reducing high cost, time, and ethical implications. Tissue-engineered (TE) *in vitro* models are rapidly developing for many applications including drug discovery, toxicity testing, and disease modelling [7]. Incorporating a porous structure into a TE model is a popular technique to overcome the diffusional mass transfer limit whilst enabling cell infiltration and integration with surrounding tissue [8]. This is an alternative to commonly used spheroidal cultures, where the porous scaffold provides a conducive environment for 3D culture. Previous studies highlight that a pore size of ~40 μm and a porosity of 90% is suitable for cell ingrowth for a variety of cell types [9,10,11], while our approach also enables the pore size and overall porosity to be tuned. This scaffold approach is open to 3D cell culture of all cell types and enables easier co-culture compared to spheroidal cultures where the use of specific cell types can be limited. In addition, the porous structure can provide a scaffold to stimulate the production of extracellular matrix (ECM) components by cells, resulting in ECM recapitulation within the model [12]. The ECM is a significant element of the tissue microenvironment and plays an integral role in maintaining normal cell function and behaviour, and can have a significant impact on cancer development [13,14].

Both natural and synthetic polymers are materials commonly used in the fabrication of TE scaffolds. Whilst natural polymers recapitulate the chemistry and architecture needed for cell attachment and growth [15], there are many associated disadvantages such as batch-to-batch variability, risk of pathogen transmission, and the potential of containing protein impurities [16,17,18]. In contrast, TE scaffolds produced from synthetic polymers are inexpensive to manufacture, possess tuneable chemical and mechanical properties, and can be reproduced accurately without the concern of polymer batch variation.

In order to incorporate porosity into a polymer-based scaffold, various manufacturing techniques have been applied, including particle leaching, solvent casting, gas foaming, and additive manufacturing, as detailed in numerous reviews [8,19,20,21,22]. Recently, high-internal-phase emulsion (HIPE) templating has become an emerging technique to produce TE scaffolds with multiple advantageous properties [23,24,25]. The scaffolds can be produced via inexpensive production methods, while the porosity can be tuned controllably. Emulsion-templated scaffolds provide high porosity and interconnectivity, enabling mass transport of nutrients and waste, cell migration, and potential vascularisation via co-culture with endothelial cells [24]. In addition, the scaffolds are highly tuneable, enabling the production of TE models for specific applications [26].

**Figure 1:**
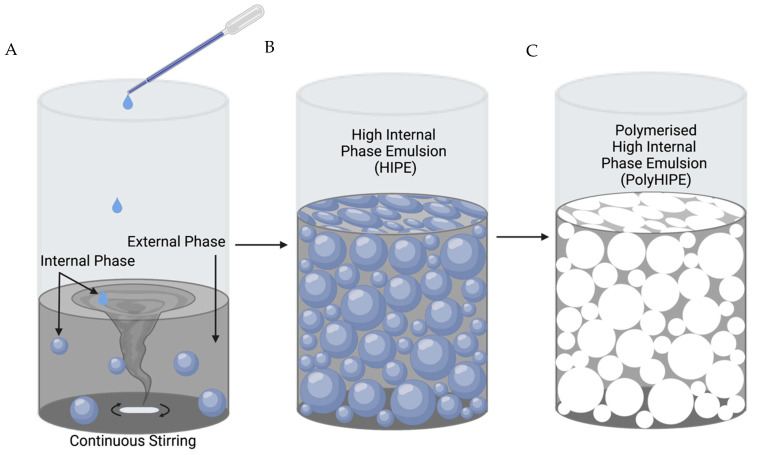
Process to fabricate polymerised high-internal-phase emulsions (polyHIPEs). (**A**) Addition of an internal phase, drop-wise, into a continuous external phase. (**B**) The external phase ruptures at the thinnest sections, transforming the internal phase into a continuous connected phase. (**C**) The external phase is solidified via polymerisation and the internal phase is removed, resulting in a porous, interconnected polymer structure. Created with BioRender.com.

Emulsion templating involves the mixing of two immiscible phases, where an internal phase (water) is dispersed within an external continuous phase (polymer) in the presence of an emulsifier, commonly a surfactant, to stabilise the emulsion (Figure 1). When the volume of the internal phase is greater than 74.05%, an emulsion is classified as a HIPE. The process of emulsion templating is well-documented in numerous studies and reviews [25,26,27,28].

Alvetex is a current gold-standard polystyrene polyHIPE-based scaffold widely used in *in vitro* assays to study cell growth, differentiation, and function [7]. Thus, the performance of novel scaffolds is often compared to Alvetex. A recent study by Aldemir Dikici et al. [23] compared Alvetex to four-arm methacrylated polycaprolactone polyHIPE scaffolds, demonstrating a similar level of performance in cell culture and ingrowth.

Polycaprolactone (PCL) has many beneficial characteristics including FDA approval, low-cost, and ease of manufacture and manipulation, as presented by Woodruff et al. [29].

In addition, the biodegradation of PCL is relatively slow (>1 year) compared to other polymers, such as polylactides (2–4 months complete resorption) and polyglycolides (weeks to months for complete resorption) [30]. Thus, it is an ideal candidate for longer-term scaffolds, implants, drug delivery applications, or testing platforms and models. PCL has a lower Young’s modulus than polystyrene (350 MPa versus 3.4 GPa), which makes it a more suitable material for soft tissue models. PCL has been used in a range of TE applications including bone [31,32], skin [33], cartilage [34], vascular [35], tendon, and ligament engineering [29].

In this study, we investigated the use of emulsion templating to manufacture a porous PCL scaffold, where high-internal-phase emulsion templating was combined with high-molecular-weight four-arm polycaprolactone methacrylate (PCL–M) to fabricate highly porous and interconnected polyHIPEs. The effect of mixing speed and post-processing washing cycles on the polyHIPEs structural and mechanical properties was investigated. The surfaces of the scaffolds were functionalised via air and acrylic acid plasma treatment and initial *in vitro* cell viability and proliferation within the scaffolds was analysed via a resazurin reduction assay. Furthermore, an *ex ovo* chorioallantoic membrane (CAM) assay was used to investigate the biocompatibility of PCL–M polyHIPEs within an *in vivo* vascularised tissue.

## 2. Materials

Photoinitiator (PI) (2,4,6-Trimethylbenzoyl Phosphine Oxide/2-Hydroxy-2- Methylpropiophenone), SLYGARD 184 Silicone elastomer base and silicone elastomer curing agent, Dulbecco’s modified Eagle media (DMEM), fetal bovine serum (FBS), penicillin/streptomycin (PS), l-glutamine, trypsin, paraformaldehyde, and resazurin sodium salt were purchased from Sigma Aldrich. Chloroform, toluene, ethanol, and methanol were purchased from Fisher Scientific. Fibronectin was purchased from YO Proteins. The surfactant, Hypermer B246, was purchased from Croda. High-molecular-weight four-arm polycaprolactone methacrylate (PCL–M, 20,000 g/mol) was synthesised in the laboratory (a general synthesis method is given in [23]).

## 3. Methods

### 3.1. Preparation of PCL–M Emulsions

Unless stated otherwise, the amounts of PCL–M (0.40 g), surfactant (0.04 g), photoinitiator (0.03 g), solvent blend (60 wt% chloroform and 40 wt% toluene, 0.60 g), and water (2 mL) were kept constant in each batch of emulsion. This resulted in an internal phase volume of 83% *w/w*. PCL–M and the surfactant were added to a glass vial, heated to dissolve the surfactant, and left to cool. Photoinitiator and the solvent blend were added to the PCL–M–surfactant mixture. The vial was protected from light and the contents were mixed (200–800 rpm) using a magnetic stirrer (20 mm × 7 mm) for 3 min at 37 °C. Once homogeneity was reached, water was added dropwise within 3 min and the emulsion was further mixed for 5 min.

### 3.2. Polymerisation of PCL–M Emulsions

Samples were either polymerised in a 2 mL syringe or in a silicone mould. For scaffolds with a 6.5 mm diameter, the PCL–M emulsion was loaded into a 2 mL syringe. Samples for mechanical testing, a dog-bone-shaped silicone mould (thickness (T): 3 mm, gage length (G): 13.5 mm, and width overall: 5.7 mm) was made using silicone elastomer base mixed with silicone elastomer curing agent (10:1 ratio). The two reagents were mixed for 5 min before being poured into a petri dish, forming a layer sufficiently covering the acrylic dog-bone, and left in an oven for 12 h to cure at 60 °C. The PCL–M emulsion was syringed into the dog-bone-shaped mould. All samples were cured for 5 min on both sides using the OmniCure Series 1000 system (100 W, Lumen Dynamics, Mississauga, ON, Canada), with 18 W/cm^2^ reported light density and spectral output from 250–600 nm. The resulting polyHIPEs were removed from the syringe or mould and washed in 100% methanol for 3 days, changing the methanol after each 24 h period. Following this, the samples were washed in water for 3 days, changing the water after each 24 h period, removing contaminants such as surfactant, solvent, and uncured emulsion. The samples were then removed from the water and left to dry in a vacuum oven at room temperature overnight.

### 3.3. Assessment of PCL–M PolyHIPEs Porosity by SEM

To observe and analyse the micro-porosity of the polyHIPE samples, scaffolds polymerised using the 2 mL syringe were sliced into 1 mm thick discs using a vibrating microtome (5100 mz, Campden Instruments, Loughborough, UK). The vibratome frequency, amplitude, and speed were set at 80 Hz, 1.50 mm, and 0.10 mm/s, respectively. The porosity and morphology of the polyHIPEs were analysed using a scanning electron microscope (SEM, Helios G4 CXe PFIB DualBeam, Thermo Fisher Scientific, Eindhoven, The Netherlands). Samples were not subject to deposition of conductive coatings (e.g., gold or carbon), in contrast to usual SEM analysis practice for polymers. To avoid surface charging and damage to the sample, a low accelerating voltage of 1 kV with typical vacuum pressure of 10^−5^ mbar at a working distance of 3 mm was applied. Working with low acceleration voltage allows for accurate visualisation of pore size and morphology of non-conductive materials such as the PCL polyHIPE substrates without the need of a metal coating [36]. An Everhart–Thornley detector (ETD) was used for low magnification images and a through lens detector (TLD) was used for high magnification images. The SEM images were used to calculate the average pore size, window size, and degree of openness. The diameter of pores and windows were measured using ImageJ v. 1.48 from the National Institutes of Health (NIH, Bethesda, MD, USA). The pores were selected by placing a 12 square grid over the image and measuring the pore diameters that were in contact with the grid. A correction factor (2/$\sqrt{3}$) was applied to adjust for the assumption that each pore was exactly bisected. The correction factor evaluates the average of the ratio R/r, where R is the actual pore diameter and r is the measured diameter of the pore, further detailed in [37]. The windows were selected by measuring any window that was found within a pore in contact with the grid. Histograms of the pore and window size were created using GraphPad (GraphPad Prism, Version 9.4.1, San Diego, CA, USA). Data points that lay outside the mean ± 3 standard deviations (1.64% of total data set) were classed as outliers and removed from the dataset. These outliers were considered to be caused by air bubbles from transfer of the emulsion to the mould.

### 3.4. Mechanical Characterisation

The elastic modulus of the PCL–M was calculated using tensile testing (MultiTest 2.5–dV Mecmesin, Slinfold, UK). The MultiTest 2.5 was equipped with 25 N and 250 N load cells that were utilised to characterise PCL–M polyHIPEs and bulk polymer, respectively. PCL–M emulsions and bulk polymer were cured in tensile test pieces and clamped between the two grips. The tensile tests were performed on each sample at a rate of 1 mm/min until the samples failed. The elastic modulus was calculated from the gradient of the initial linear region of the stress–strain curve for each sample. The ultimate tensile strength was measured at the point at which the samples withstood the maximum stress. Maximum elongation was the defined as the percentage elongation at the time the samples broke.

### 3.5. Surface Wettability of PCL–M polyHIPE

Water contact angle measurements were used to analyse and quantify the hydrophilicity of PCL–M bulk and polyHIPE disc surfaces. A disc silicone mould was made using silicone elastomer base mixed with silicone elastomer curing agent (10:1 ratio). The two reagents were mixed for 5 min before being poured into a petri dish, forming a layer sufficiently covering acrylic discs, and left in an oven at 60 °C for 12 h to cure. PCL–M bulk discs were produced by heating PCL–M in a glass vial until the polymer melted. Then, 5 wt% of PI was added to the PCL–M and thoroughly mixed. The discs were injected into the silicon mould and cured on both sides for 5 min using the OmniCure Series 1000 system. The discs were treated with air and acrylic acid plasma for durations of 2 and 30 min, respectively. The sessile drop method with deionised water was used to measure the contact angle on the functionalised polyHIPE and bulk PCL–M discs (diameter 6.5 mm and 15 mm, respectively) using a contact angle goniometer (Goniometer FTÅ 200) paired with First Ten Angstroms (FTA) software. The mean reported from each variable was acquired from three surface locations.

### 3.6. Assessment of Surface Functionalisation of PCL–M Scaffolds

X-ray photoelectron spectroscopy (XPS) was used to assess the surface functionalisation of PCL–M polyHIPE scaffolds following plasma coating with air and acrylic acid and/or fibronectin coating. Samples were analysed using a Kratos AXIS Ultra DLD instrument (Department of Chemistry, the University of Sheffield, UK). Spectra were recorded using a monochromatized Al Kα X-ray source (1486.6 eV) operating at a power of 150 W. An internal flood gun was used to reduce the charging of the sample during irradiation. Each sample was analysed at an emission angle normal to the sample surface. Data processing, analysis, and charge correction were carried out using Casa XPS software (Casa Software Ltd., Teignmouth, UK). Component peaks within the recorded C(1s) spectra were deconvoluted and fitted to an asymmetric Lorentzian line-shape (model LA with parameters α = β = 1.53 and m = 243). The aliphatic hydrocarbon component of the C(1s) was set to 285.0 eV as an internal reference.

### 3.7. General Cell Culture

MDA-MB-231 cells (triple-negative breast cancer cell line) [38] were used to evaluate the proliferation and morphology of cancer cells with PCL–M polyHIPE scaffolds. The MDA-MB-231 cells were purchased from Merck (ECACC) and transduced to express luciferase2 and mStrawberry by transfection with a transposon and the transposase PiggyBac using methodology developed previously [39]. The cells were transduced and selected with puromycin, stocks frozen within 5 passages, and then used within 30 passages of receipt from ECACC. The cells were thawed, transferred to media (DMEM supplemented with 10% FBS, 1% PS, 1% L-glutamine), and centrifuged at 95 g for 5 min. The cell pellet was resuspended in fresh media with 1 μg/mL puromycin and cultured until 90% confluence with media changes every 3 days. Puromycin was removed from the media 24 h before each experiment.

### 3.8. Scaffold Fabrication for Cell Culture

To initially characterise cell–scaffold interactions, polyHIPE discs (6 mm diameter and 1 mm depth) were used. To sterilise, all scaffolds were washed in methanol followed by PBS. Scaffolds were treated for 24 h before cell culture using air plasma or acrylic acid (AAc) plasma (in house set-up as described in [40]). Air plasma was applied to the scaffold discs with a power of 50 W for 2 min. AAc plasma was applied to the scaffold discs with a power of 10 W for 30 min. Following this, the scaffolds were placed in a 24 well-plate and soaked in one of two conditions; phosphate-buffered saline (PBS) or fibronectin (10 µg/mL) for 12 h in an incubator at 37 °C and 5% CO_2_.

### 3.9. MDA-MB-231 Cell Seeding on PCL–M polyHIPE Scaffolds

Once reaching 90% confluency, cells were detached from the cell culture flask using trypsin. After 4 min, the trypsin was neutralised with cell culture media (ratio of 1:2), followed by centrifugation (95× *g* for 5 min), and resuspended in fresh media before counting using the trypan blue exclusion method to assess cell viability. The pre-soaking solutions were removed from the scaffolds and 25 µL of MDA-MB-231 cells at $1\times 10^{6}\;\mathrm{cells}/\mathrm{mL}$ were transferred onto each scaffold and left for 1 h in the incubator (37 °C and 5% CO_2_) to allow cell attachment. After 1 h, a further 25 µL of MDA-MB-231 cells at $1\times 10^{6}\;\mathrm{cells}/\mathrm{mL}$ were transferred onto the second side of the scaffolds and left for an additional 1 h in the incubator. After 1 h, fresh media was placed in each well and incubated for 7 days.

### 3.10. Cell Viability on PCL–M polyHIPE Scaffolds

The viability of cells on the scaffold was assessed using the resazurin reduction (RR) assay. For this, 1 mM resazurin stock solution was diluted in cell culture media to form a 10% *v/v* resazurin working solution. The media was removed and discarded from each well and a further 1 mL of the working solution was added to each well. The well plate was protected from light and incubated for 4 h at 37 °C. An orbital rocker (30 rpm) was used in the incubator to ensure full penetration of the resazurin working solution. A total of 200 µL was taken, in triplicate, from each scaffold and transferred to a 96 well-plate. A spectrofluorometer (BioTek ELx800, Agilent BioTek, Santa Clara, CA, USA) was used to read the fluorescence of each well at an excitation wavelength of 540 nm and an emission wavelength of 630 nm. The working solution was removed from the scaffolds and each scaffold was further washed with PBS twice before adding fresh cell culture media and continuing incubation. The assay was performed at day 1 and repeated at day 3 and 7.

### 3.11. CAM Assay

The *ex ovo* chick chorioallantoic membrane (CAM) assay as described by Ramos-Rodriquez et al. [33] was used to study potential toxic effects of the samples within a developing vascular system. Briefly, pathogen-free fertilised eggs (Gallus domesticus), obtained from Henry Stewart & Co. (Fakenham, UK), were cleaned with 20% *v/v* industrial methylated spirits (IMS) and incubated in a humidified hatching incubator (Rcom King Suro Max-20, P&T Poultry, Powys, Wales, UK) at 38 °C for 3 days. On day 3, the eggs were cracked into sterile 100 mL weighing boats with 3 mL of PBS + 1% *v/v* penicillin–streptomycin solution (100 IU/mL–100 mg/mL). The eggs were further incubated at 38 °C in a humidified cell culture incubator (Binder, Tuttlingen, Germany). On day 7, the sterilised 200 µm sectioned polyHIPE discs were implanted within the boundaries of the CAM and incubated for a further 5 days. On day 11, the CAM was imaged using a digital camera and MicroCapture software (version 2.0). Moisturising cream (Lacura, Atherstone, UK) was injected into the surrounding area of the sample to provide contrast between blood vessels and the sample. The vascular density of the CAM was further analysed from the images using the vessel analysis ImageJ plugin. Following imaging, all embryos were sacrificed by the end of day 11 of embryonic development. All embryos were incubated and handled under the guidelines of the Home Office, UK.

### 3.12. Statistical Analysis

Statistical analysis was carried out using analysis software GraphPad Prism (Version 9.4.1, San Diego, CA, USA). All data was analysed using one-way or two-way analysis of variance (ANOVA) followed by Games–Howell (*n* > 50) and Dunnett T3 (*n* < 50) multiple comparisons tests. Error bars on graphs indicate standard deviation and all *n* values are given in figure captions where relevant.

## 4. Results

### 4.1. Manufacturing and Assessment of PCL–M polyHIPEs Porosity

**Figure 2:**
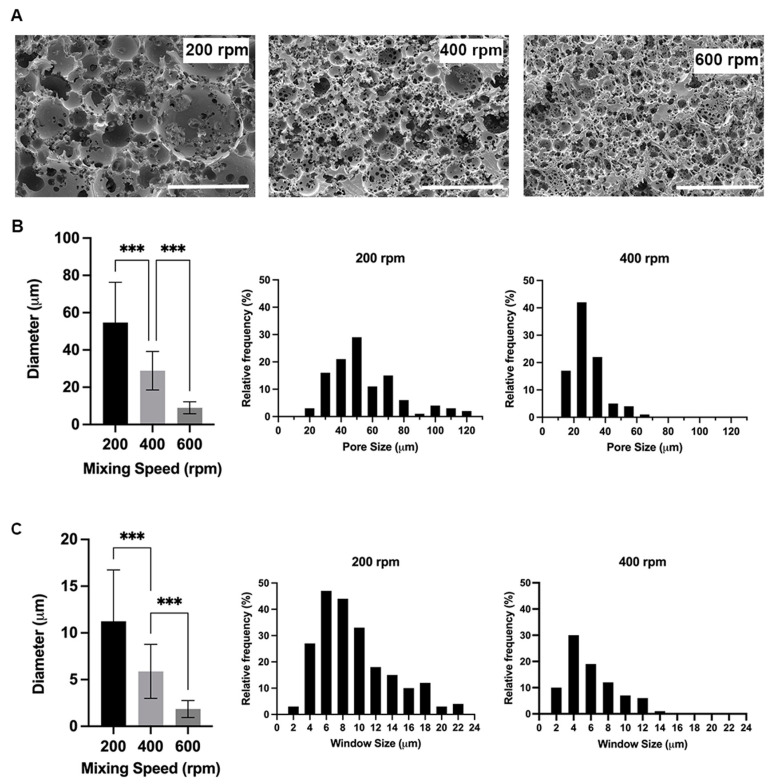
PCL–M polyHIPEs fabricated using different mixing speeds. (**A**) SEM images demonstrating the changes in polyHIPE pore size and morphology due to varying mixing speed (scalebar represents 100 μm). The addition of the internal phase to the external phase affects (**B**) the pore and (**C**) window size (mean +SD, *n* = 72 and 82, respectively, *** *p* < 0.001), and the relative frequency and distribution of pore and window sizes.

The ratio of the volume of the internal phase (water) to the total volume (water and polymer) results in a polyHIPE scaffold with an internal phase volume of 83%. The porosity of the PCL–M polyHIPEs is greatly affected by the mixing speed used during the manufacturing of the emulsion. The mean diameter of pores (D) within the polyHIPE significantly decreases from 55 ± 22 µm at 200 rpm to 29 ± 10 µm at 400 rpm (Figure 2A). Following a similar trend, there is a further significant decrease in the mean pore diameter from 400 rpm to 9 ± 3 µm at 600 rpm. Following 600 rpm, any increase in mixing speed does not result in a significant decrease in pore size. However, the structure of the pores is affected, with the morphology of the pores becoming distorted and disorganised. Similarly, the window size within the polyHIPEs decreases with increasing mixing speed, with a significant decrease in mean window diameter (d) from 200 rpm to 400 rpm to 600 rpm (11 ± 5 µm, 6 ± 3 µm, and 2 ± 1 µm, respectively) (Figure 2B). The literature describes the fact that pore sizes >10 µm are required for cell attachment and infiltration [41]. Thus, further analysis of pores was limited to scaffolds fabricated at 200 and 400 rpm.

High variability is observed in pore and window size in scaffolds when mixing at 200 and 400 rpm. Whilst the average pore sizes are measured at 55 ± 22 µm and 29 ± 10 µm at 200 and 400 rpm, respectively, the pores range from 22–117 µm and 12–63 µm, respectively (Figure 2B). Furthermore, the interconnections between pores averages 11 ± 5 µm and 6 ± 3 µm; however, they range from 3–26 µm and 2–14 µm for 200 and 400 rpm, respectively (Figure 2A,B). The degree of interconnectivity (d/D) is not affected by changing the mixing speed and remains at 0.2.

### 4.2. Mechanical Characterisation of PCL–M polyHIPEs

**Figure 3:**
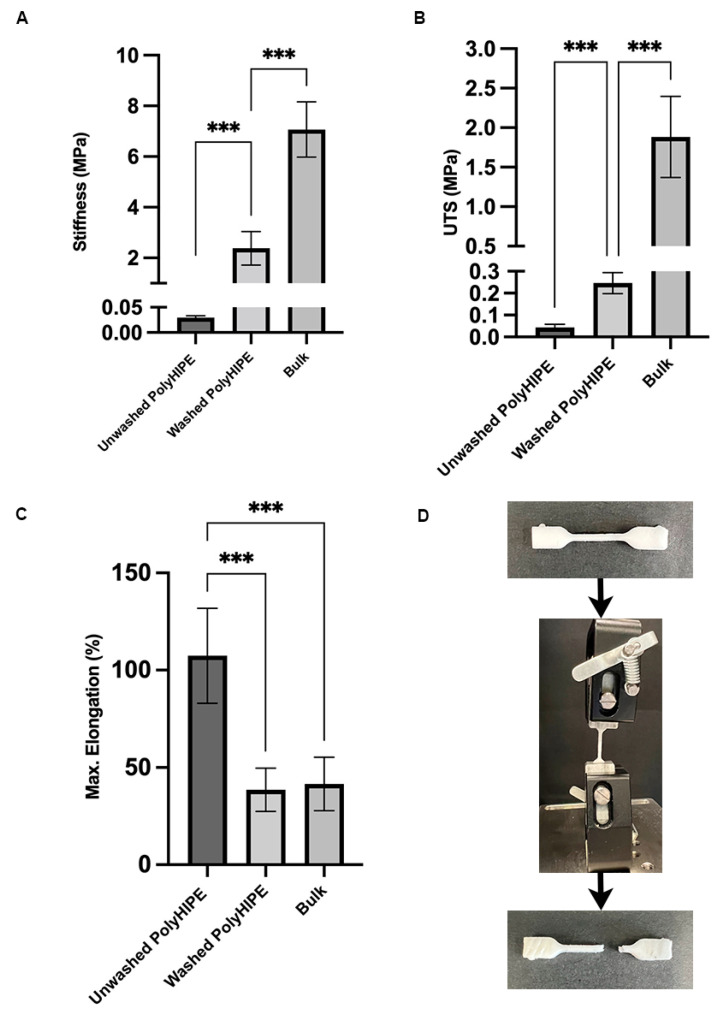
Mechanical properties of PCL–M polyHIPEs under two conditions (washed and unwashed) and bulk PCL–M. (**A**) Stiffness, (**B**) ultimate tensile strength (UTS), and (**C**) maximum elongation of the polyHIPE and bulk scaffolds (mean +SD, N = 3, *n* = 5, *** *p* < 0.001). (**D**) A polyHIPE sample before and after tensile testing, demonstrating the common region of failure when the material broke.

Tensile tests were conducted on PCL–M polyHIPE under both washed and unwashed conditions and on bulk PCL–M. The stiffness is measured at 0.03 ± 0.01 MPa, 2.38 ± 0.66 MPa, and 7.07 ± 1.09 MPa for unwashed PCL–M polyHIPE, washed PCL–M polyHIPE, and bulk PCL–M, respectively (Figure 3A). The ultimate tensile strength increases from the unwashed to washed polyHIPE scaffold and then further increases for bulk PCL–M, measuring 0.04 ± 0.02 MPa, 0.25 ± 0.05 MPa, and 1.88 ± 0.51 MPa, respectively (Figure 3B). The maximum elongation of the scaffolds decreases significantly from unwashed to washed polyHIPE (107 ± 24% and 39 ± 11%, respectively) (Figure 3C). However, there is no significant change in maximum elongation observed between washed polyHIPE scaffolds and bulk PCL–M (39 ± 11% and 42 ± 14%, respectively). Therefore, the washing process of the PCL–M polyHIPE scaffolds has a significant effect on the mechanical properties of the material.

### 4.3. Effect of Washing

**Figure 4:**
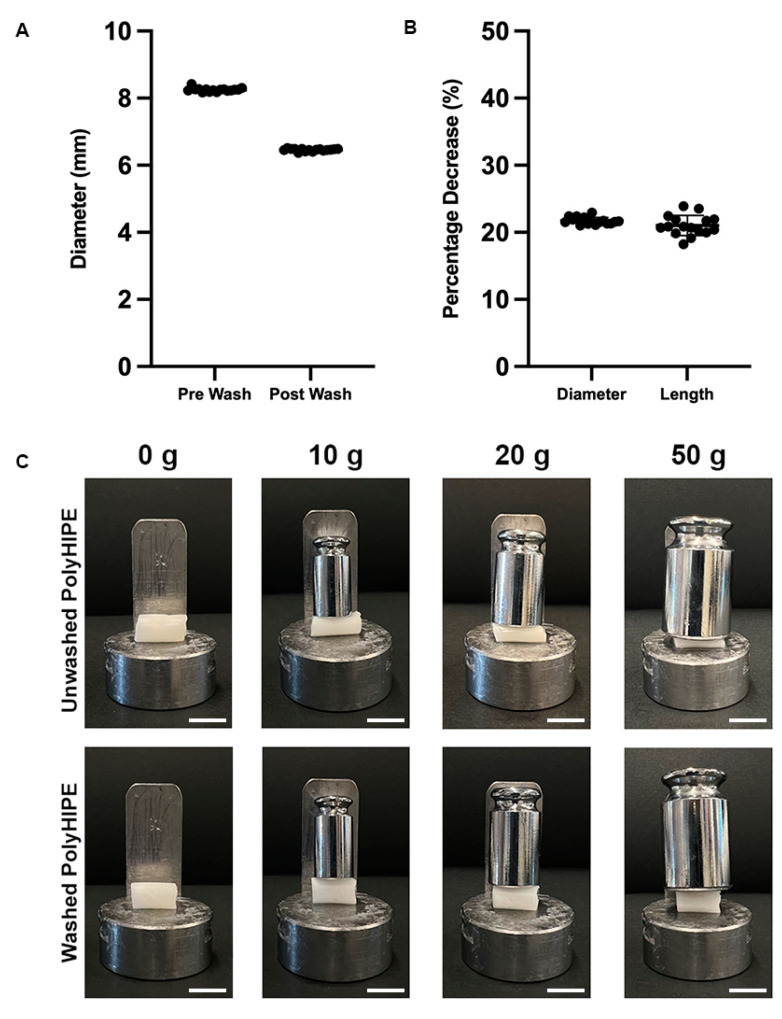
The effects of the post-processing washing cycles on PCL–M polyHIPEs, (**A**) the decrease in diameter and (**B**) the percentage decrease in diameter and length of cylindrical scaffolds (*n* = 16, black dots represent data points). (**C**) The significant changes in stiffness of PCL–M polyHIPEs following post-processing washing cycles could be visually observed when loads ranging from 0–50 g were applied (scale bar = 1 cm).

The polyHIPE scaffold must undergo post-processing washing cycles to remove remaining solvent, surfactant, and initiator before they are further utilised for cell culture. The washing cycle is observed to affect the size of the scaffolds, resulting in a significant decrease in scaffold diameter from 8.0 mm to 6.5 mm (Figure 4A). A 20% decrease is measured in both scaffold diameter and length, demonstrating that the effect of washing on the polyHIPE scaffolds is isotropic (Figure 4B). The compressive stiffness of the unwashed samples was unable to be quantified as the value was under the detection limit for a 25 N strain gauge. However, a significant increase in stiffness post-washing is clearly observed using calibrated weights (Figure 4C).

### 4.4. Surface Wettability of PCL–M polyHIPE

**Figure 5:**
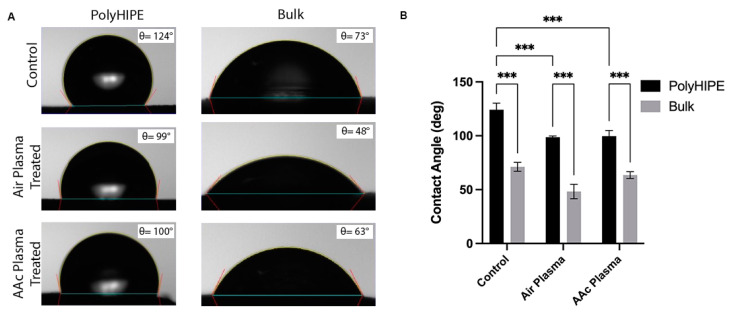
(**A**) The effect of air and acrylic acid plasma treatment on the contact angle of polyHIPE and bulk PCL–M surfaces. (**B**) The mean ± SD of the effect air and AAc plasma treatment on the wettability of polyHIPE and bulk PCL–M (*n* = 3, *** *p* < 0.001).

The surfaces of both washed PCL–M polyHIPEs and bulk PCL–M were functionalised using air plasma and acrylic acid (AAc) plasma treatment. Under all conditions (control, air, and AAc plasma treatment), bulk PCL–M is more hydrophilic than the comparative PCL–M polyHIPE scaffolds. There is a decrease in contact angle from polyHIPE to bulk PCL–M of 53°, 51°, and 37° across the three conditions; control, air, and AAc plasma treatment, respectively (Figure 5A). Both air and AAc plasma treatment result in a significant decrease in surface contact angle for PCL–M polyHIPEs (Figure 5B), with air plasma treatment yielding the most effective reduction in surface contact angle for both PCL–M polyHIPEs and bulk PCL–M from 124° ± 6° and 71° ± 4° to 99° ± 6° and 48° ± 7°, respectively.

### 4.5. Surface Functionalisation of PCL–M Scaffolds

The polyHIPE scaffolds were also analysed via X-ray photoelectron spectroscopy (XPS). The survey scan reveals 24 at% oxygen and 76 at% carbon for the four-arm caprolactone methacrylate, close to the theoretically expected ratio of 25/75 at%. Air plasma coating introduces a small amount of nitrogen on the surface (~1%), while the carbon to oxygen ratio remains mainly unchanged for both air and acrylic acid coating. Importantly, all fibronectin-coated samples exhibit an increased amount of nitrogen on the surface, from 3.25 at% nitrogen for a non-plasma-coated PCL–M surface, to 5.32 at% for an air-plasma-treated surface and 6.33 at% for an acrylic-acid-plasma-treated surface. This indicates that the protein coating present on all fibronectin-coated surfaces has an affect while acrylic acid and air plasma increase protein attachment. A high-resolution scan of the carbon 1s region reveals a change in surface functional groups depending on the treatments highlighted in Table 1. Combining the information from the survey and high-resolution scans reveals the following notable trends; (i) both air and acrylic acid treatment increase the amount of hydroxyl and carboxyl surface moieties, while (ii) both amine and amide moieties are observed when coating with fibronectin.

### 4.6. Interaction of PCL–M polyHIPEs with a Vascular Network Using an Ex Ovo CAM Assay

**Figure 6:**
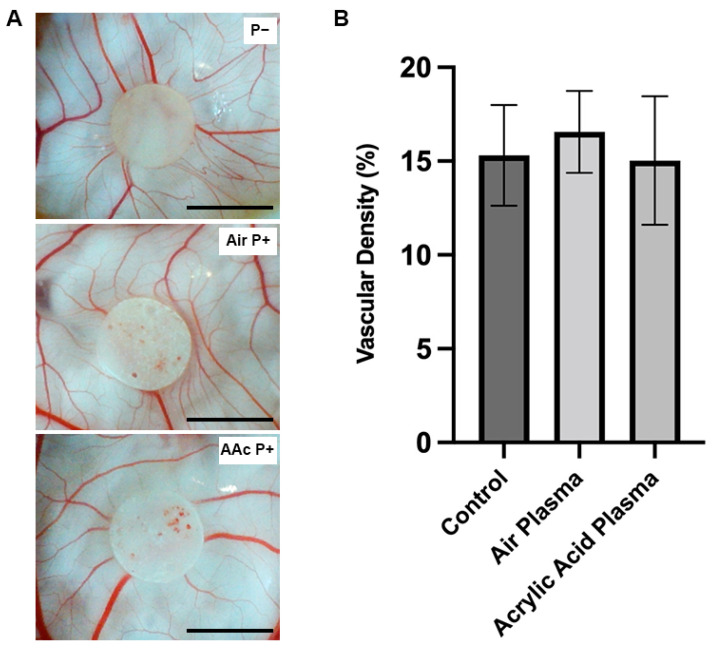
Assessment of PCL–M polyHIPE biocompatibility. (**A**) Images of PCL–M polyHIPEs functionalised with air and acrylic acid plasma treatment on chorioallantoic membrane (CAM) at day 11 (scale bar represents 5 mm). (**B**) The vascular density of the vessels surrounding the polyHIPE scaffolds (*n* = 5).

The CAM assay is an established *ex ovo* model able to assess the initial interactions of a biomaterial within a well-established vascularised tissue [33,42,43,44]. The assay investigated the biocompatibility of PCL–M polyHIPEs with and without surface functionalisation via air and AAc plasma treatment (Figure 6A). There is no significant change in vessel density surrounding the polyHIPE scaffolds (Figure 6B).

### 4.7. Activity and Interaction of MDA-MB-231 Cells on PCL–M Scaffolds

**Figure 7:**
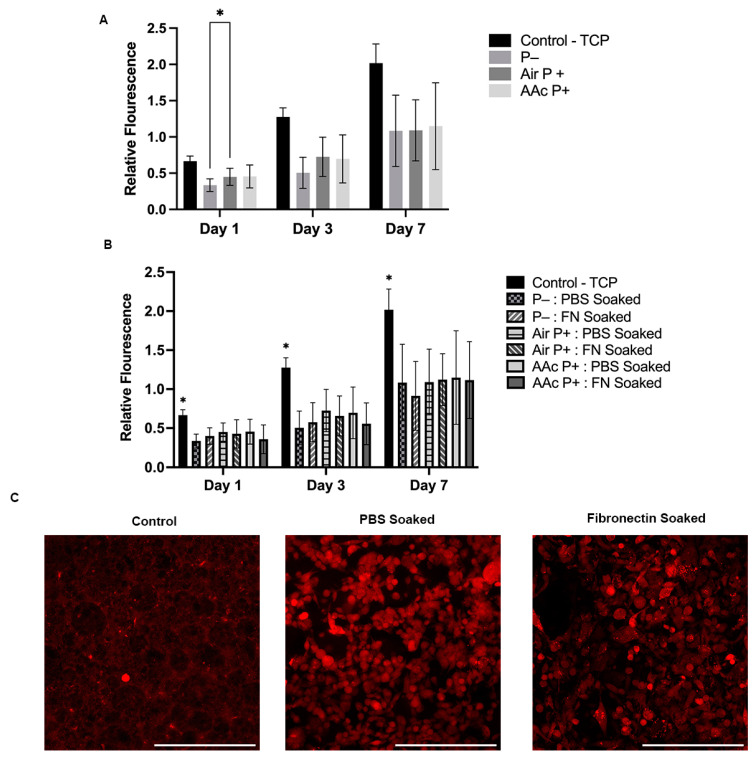
Biological assessment of PCL–M polyHIPEs. The metabolic activity of MDA-MB-231 cells via a resazurin reduction assay across 7 days on (**A**) untreated PCL–M scaffolds (P−) or scaffolds functionalised with air and acrylic acid (AAc) plasma treatment (P+) and (**B**) preconditioned with fibronectin (N = 3, *n* = 5, * *p* < 0.033). (**C**) Confocal images of MDA-MB-231 cells transduced with mStrawberry following 7 days cultured on PCL–M scaffolds pre-soaked in PBS or fibronectin (scalebar represents 200 μm).

A seven-day study using Resazurin reduction was used to determine the metabolic activity and cell proliferation on PCL–M scaffolds. PCL–M scaffolds under three different plasma treatment conditions were analysed. The results show a consistently significant increase in metabolic activity between the control and three treatment conditions through days 1, 3, and 7 (Figure 7A). There is a significant difference at day 1 between untreated scaffolds (P−) and the air-plasma-treated scaffolds (air P+). At all other time points and between all conditions, P−, air P+, and acrylic acid plasma treatment (AAc P+), there is no significant difference between metabolic activity. However, air P+ and AAc P+ scaffolds show slightly increased metabolic activity at day 1 and 3 compared to P− scaffolds. Also, via comparison to the 2D control at the day 1 timepoint of the resazurin reduction assay, the adhesion of MDA-MB-231 cells can be approximated. Following seeding, approximately 50% of cells adhere to P− scaffolds and 68% adhere to air and AAc P+.

Furthermore, the effect on metabolic activity and cellular interaction by coating the surfaces with fibronectin was investigated. A seven-day resazurin reduction assay measures no significant difference between the fibronectin-soaked scaffolds and the control PBS-soaked scaffolds through all plasma treatment conditions (Figure 7B). At day 7, the samples were fixed and further analysed via confocal. As shown in the images, there is little difference in the presence and morphology of MDA-MD-231 cells observed on scaffolds that are soaked in fibronectin compared to scaffolds that are PBS soaked (Figure 7C). Low background autofluorescence is observed on the control cell-free scaffold, with a small number of brighter auto-fluorescent artefacts.

## 5. Discussion

An open porous interconnected architecture is vitally important within scaffolds for tissue engineering applications. Interconnections through pores provide transport channels for cell migration, mass transport of cell nutrients and waste, and support cell signalling [45,46]. Open-surface porosity is also important for cell ingrowth, which is affected by the surface of the polyHIPE when it is in contact during photopolymerisation. Contact with a mould can cause a reduction in open porous morphology at the interfaces of the emulsion and the moulds [32]. Therefore, to utilise the inner open, interconnected porous morphology of the polyHIPE structure, it was decided to section bulk cylinders of polyHIPE, as described in Section 3.3.

When investigating the effect of mixing speed on polyHIPE structure, a reciprocal relationship is observed, as the mixing speed increases the pore sizes, and window sizes decrease (Figure 2A,B), in line with previous studies [26,27,47,48,49]. These findings demonstrate the ability to easily tune the internal structure of polyHIPEs via simple manufacturing adjustments. A high variability in both pore and window size is observed in the PCL–M polyHIPE scaffolds when 200 and 400 rpm mixing speeds are used as the internal phase (water) is added (Figure 2B).

A dynamic range in pore size is beneficial as it can provide multiple elements necessary to form a functioning biomimetic scaffold. It has been reported that micro-pores (100 nm–5 µm) enable cell attachment, medium-size pores (5–250 µm) enable cell infiltration, whilst macro-pores (>250 µm) support neo-vascularisation and, thus, scaffold vascularisation [46,50,51]. The larger interconnections provide micro-features and further support cell infiltration and transport of nutrients and waste through the scaffold. Smaller interconnections provide nano-features, with Smith et al. [46] reporting enhanced cell attachment and ECM formation within scaffolds.

Moreover, Bružauskaitė et al. [52] demonstrate that smaller pores are required for smaller cell types (e.g., fibroblasts) to enhance cell attachment by reducing unwanted cell migration. In addition, large pore sizes can reduce intracellular signalling [53], further demonstrating the importance of being able to tune the porosity of a chosen scaffold to the required application.

As described by Poltavets et al. [54], it has been identified that cancer cell behaviour is driven by biomechanical signals of the tumour microenvironment. Within breast cancer tissue, the presence of organised collagen fibres results in an increase in tissue stiffness compared to surrounding tissue, enhancing tumour progression and metastasis [55,56]. The Young’s modulus of the material is measured at 2.38 MPa, resulting in stiff scaffolds that are easily handled. The scaffolds provide an environment in which cells can lay down ECM with a stiffness suitable for the specific cell type. To enhance this process, the scaffolds could be combined with a hydrogel substrate within the structure, to provide a predefined ECM surrounding the cells [57].

Surprisingly, the stiffness of the washed PCL–M polyHIPE, which has an internal phase volume of 83%, is only three-fold less than bulk PCL–M, demonstrating the advantageous characteristics of using a high-molecular-weight PCL–M polyHIPE structure, achieving relatively high stiffness with high-internal-phase volumes. On the other hand, the ultimate tensile strength (UTS) of the polyHIPE scaffold compared to the bulk material decreases nearly eight-fold from bulk to washed polyHIPE scaffolds. Interestingly, the structure of the polyHIPE has no significant effect on the maximum elongation of the scaffolds when compared to the bulk material.

During the post-processing of the polyHIPEs, methanol and water washing cycles were used. These washing stages are important to remove remaining surfactant, photoinitiator, and solvent from the scaffold. Interestingly, during the process it is observed that the post-processing affects the structural and mechanical properties of the scaffolds (Figure 4C). Before washing, the scaffolds show higher elasticity, while scaffold stiffness is lacking. Post-washing, the maximum elongation halves but there is an 80-fold increase in stiffness. In addition, isotropic shrinking of the scaffold by 20% is observed. Through the washing process, this change in stiffness and size is most noticeable following the water wash cycle compared to the methanol wash cycle. Thus, it is deduced that the change in properties occurs due to the removal of excess solvent within the scaffold, which act as a plasticiser of the produced thermoset polymer construct. This finding corresponds well with the findings of Dikici et al. [42]. The study used ethanol to expand a PCL polyHIPE tube. After insertion of an electrospun layer, a PBS wash was used to remove excess solvent, resulting in shrinkage of the polyHIPE tube. This also indicates that the mechanical properties can be used as a simple test for the efficiency of solvent removal.

In previous studies, the significant impact of dry and wet conditions on the mechanical properties of polymer scaffolds was reported, demonstrating a significant decrease in stiffness, maximum elongation, and UTS from dry to wet conditions [32,58,59]. As tissue-engineered constructs and models are commonly used within fluidic systems to recapitulate *in vivo* conditions, tensile tests were conducted using wet polyHIPE samples in this study.

One of the most significant disadvantages of using PCL in tissue engineering constructs is its hydrophobicity. The degree of hydrophobicity is observed to significantly increase from bulk to polyHIPE PCL–M (Figure 5). These findings correspond to the Wenzel model, which describes how surface roughness enhances hydrophobicity characteristics due to the chemistry of the surface [60]. Thus, if a material is hydrophobic, surface roughness enhances the degree of hydrophobicity further. The micropores within the surface of the polyHIPE discs increase the surface roughness, therefore, enhancing the degree of hydrophobicity of the surface compared to bulk PCL–M.

Many studies functionalise the surface of materials using a range of plasma treatments to produce more hydrophilic surfaces. Far et al. highlighted an increase in hydroxyl moieties on polyglycerol sebacate (PGS) surfaces after air plasma treatment, which was correlated with a reduction in water contact angle [61]. The results from this study concur with these findings, identifying significant reductions in surface contact angle when treating the PCL–M polyHIPE with air or acrylic acid plasma. Furthermore, plasma polymerisation of a surface is a common technique used to produce biomaterials with chemically reactive surfaces, which improve cell proliferation, and interacts and permanently binds with biologically active molecules [62,63]. Depending on the specific plasma treatment utilised, high concentrations of specific functional groups are deposited on the surface of the substrate [61,64]. Different functional groups interact differently with biological molecules and cells. For example, Cools et al. [65] observed a positive effect on the generation of glycosaminoglycans matrix when plasma coating via helium and acrylic acid; however, acrylic acid plasma treatment further stimulated cell migration through scaffolds.

Air and acrylic acid plasma treatments lead to the formation of hydroxyl and carboxyl functional groups, respectively, on the surface of the treated material [64], which is also confirmed by the high-resolution XPS results (Table 1). Plasma treatment clearly decreases the amount of aliphatic carbon (R/H-C) and increases the amount of oxidised carbon moieties (C-OR and COOH) at the surface. Functionalisation via the formation of hydroxyl and carboxylic groups are two of the most common methods used within biomedical applications [66,67]. Thus, the effect of air and acrylic acid plasma treatments on cell and biological molecule (fibronectin) interaction was investigated. The high-resolution XPS scan reveals there is an increase of up to 5 at% of the 288.2 eV peak, which can be attributed to the amide carbon, and is a clear indication of protein binding. Surprisingly, there is no significant difference in cell metabolic activity when pre-soaking the scaffolds in fibronectin compared to PBS. In addition, there is no significant difference in cell metabolic activity between the air- and acrylic-acid-treated scaffolds, and the control, non-treated scaffolds. However, similar findings with air plasma treatment were published by Aldemir Dikici et al. [32] for PCL polyHIPE scaffolds.

Furthermore, there is little difference in the cell morphology on the surface of scaffolds when pre-soaked with PBS compared to the same scaffolds pre-soaked with fibronectin (Figure 7C). Overall, the metabolic activity of the cells on the scaffolds increases approximately three-fold in 7 days, indicating that PCL–M polyHIPE scaffolds are viable options for cell growth and proliferation. Rabionet et al. [68] observed similar findings when culturing MDA-MB-231 cells on electrospun PCL scaffolds, additionally presenting improved metabolic activity on electrospun scaffolds when increasing the pore area from 0.24 µm^2^ to 0.84 µm^2^. Furthermore, fibronectin-soaking and/or plasma-treating the scaffolds did does yield any further improvement in cell adhesion or proliferation compared to untreated PCL–M polyHIPE scaffolds.

The CAM assay is an established *ex ovo* model used within many studies to determine cell infiltration and material capability to support vascularization. In addition, the CAM assay has been documented as a tool to investigate material biocompatibility [69,70], with Ribatti et al. [71] describing the CAM assay as an integral part of biocompatibility testing process for the development of biomaterials. Mangir et al. [44] reported that average embryo survival rates for intermediate users is 68%. In this study, the embryo survival rate is 69%, demonstrating the material does not have an adverse effect on embryo survival. In conjunction with the resazurin reduction assay, demonstrating increasing cell metabolic activity across 7 days, PCL–M polyHIPEs can be classed as biocompatible. Furthermore, the consistency in vessel density surrounding the polyHIPE scaffolds demonstrates that PCL–M scaffolds are bioinert regardless of surface functionalisation via air and acrylic acid plasma treatment. Importantly, surface functionalisation via air and AAc plasma treatment does not adversely affect scaffold biocompatibility, while not greatly enhancing the vessel or cell growth either, identifying that this processing step could be omitted for these PCL-based polyHIPE scaffolds.

Whilst the current model uses a porous scaffold to increase the diffusional mass transfer limit and enhance cell attachment, advancements in the model could include vascularisation of a polyHIPE scaffold by co-culturing with endothelial cells [42] within an active perfusion system, as previously successfully demonstrated [72]. Pore sizes around 250 µm have been reported to be suitable for vascularisation [73]. However, there are a number of studies that report successful vascular invasion within smaller pore sizes. Chiu et al. [74] demonstrated no significant difference in vascular invasion after 3 weeks into the bulk of scaffolds with pore sizes ranging from 100–150 µm and 50–100 µm. Moreover, Artel et al. [75] show that vascularisation to the centre of a porous polymer scaffold can occur through pores ranging 40–270 µm in diameter, however, the time for vascularisation to the centre increases as average pore diameter decreases. Thus, these findings would suggest both the PCL–M polyHIPEs manufactured with mixing speeds of 200 and 400 rpm could be suitable candidates for future *in vitro* vascularisation models.

## 6. Conclusions

In this study, we fabricated a polyHIPE scaffold using photocurable high-molecular-weight four-arm PCL–M. By altering the mixing speed during emulsion fabrication, we demonstrate that the structural properties of the resulting polyHIPE can be tuned for a specific application and cell type. Lower mixing speeds (200 and 400 rpm) produce scaffolds with larger pores and interconnections, which are a more suitable environment for cell adhesion, infiltration, and vascularisation. Interestingly, whilst the polyHIPE structure provides a high-internal-phase volume, the mechanical properties are relatively comparable to bulk PCL–M. Surface functionalisation of the polyHIPEs via plasma treatment and fibronectin adsorption shows little improvement in cell adhesion and morphology. Furthermore, this study demonstrates the biocompatibility and bioinert properties of PCL–M polyHIPEs regardless of surface modifications via fibronectin adsorption and/or air and acrylic acid plasma treatment. In conclusion, we demonstrate that high-molecular-weight PCL–M polyHIPE is a good candidate for TE scaffolds with potential for vascularisation and active perfusion.

## Figures and Tables

**Table 1:** High-resolution XPS scan data for the different surface treatments, (i) untreated, (ii) air-plasma-treated, (iii) acrylic-acid-plasma-coated, (iv) fibronectin-coated, (v) air-plasma-treated and fibronectin-coated, and (vi) acrylic-acid-plasma-coated and fibronectin-coated.

C1s	C-C/C-H at% (285 eV)	C-O/C-N at% (286.2 eV)	(C,N)-C=O at% (288.2 eV)	HO-C=O at% (288.9 eV)
Untreated	67.6 ± 0.1	19.1 ± 0.3	2.1 ± 0.2	11.3 ± 0.1
Air plasma	62.8 ± 3.0	22.4 ± 2.4	1.8 ± 0.4	13.1 ± 1.0
AAc plasma	56.3 ± 1.7	28.0 ±2.0	1.2 ± 0.1	14.6 ± 0.2
Untreated and fibronectin	61.4 ± 0.3	23.6 ± 0.3	4.1± 0.2	10.9 ± 0.9
Air plasma and fibronectin	52.7 ±2.6	29.3 ± 2.7	6.1 ± 0.1	11.8 ± 0.1
AAc plasma and fibronectin	55.6 ± 0.4	28.7 ± 0.3	6.2 ± 0.6	9.6 ± 0.5

## Data Availability

Data will be available upon request.
